# Addressing historical biases and the limits of biomedical commodities in vector-borne disease control in Africa

**DOI:** 10.1371/journal.pgph.0006840

**Published:** 2026-07-14

**Authors:** Jennifer S. Lord, Ellie Sherrard-Smith, Antoine Sanou, Fredros Okumu

**Affiliations:** 1 Department of Vector Biology, Liverpool School of Tropical Medicine, Liverpool, United Kingdom; 2 Université Yembila Abdoulaye TOGUYENI, Fada N’Gourma, Burkina Faso; 3 Centre National de Recherche et de Formation sur le Paludisme, Ouagadougou, Burkina Faso; 4 School of Biodiversity, One Health and Veterinary Medicine, University of Glasgow, Glasgow, United Kingdom; 5 Environmental Health and Ecological Sciences Department, Ifakara Health Institute, Ifakara, Tanzania; Institute of Tropical Medicine: Instituut voor Tropische Geneeskunde, BELGIUM

## Abstract

In this essay, we describe efforts to curtail the spread of vector-borne infections and how these efforts are entangled in the causes and consequences of the destruction of life, and the ecosystems which support life, on our planet. Using examples from malaria and sleeping sickness, we show how current efforts to control vector-borne diseases reflect the Western logic of a highly compartmentalized, hierarchical approach, dominated by biomedicine and commoditized interventions. These have dramatically reduced morbidity and mortality yet perpetuate colonial legacies, global inequities, and environmental harm. For instance, billions of insecticide-treated nets, rapid diagnostic tests, and other single-use plastics used in health products generate waste and emissions, while reinforcing fossil-fuel dependence and Global North dominance. These approaches treat diseases as isolated problems detached from the broader socio-ecological system. They excel at suppressing symptoms but leave untouched the root causes such as poverty, ecosystem destruction, and entrenched power imbalances. Current disease research and control strategies are situated within a wider dominant culture that has led to multiple undesirable effects on our planet (including climate, ecological functions, and biosphere integrity) driven largely by wealthy minorities and a global extractive economic system. Our intention is not to promote the idea that communities should be denied commodities that protect against vector-borne disease, but to identify that a shift is urgently required to make space for alternatives. A change involving transdisciplinary, socially-just strategies, that center local leadership, and enforce full life-cycle responsibility for health commodities; all of which are essential to safeguard both human and planetary health.

## Introduction

In this essay, we describe efforts to curtail the spread of vector-borne infections and how these efforts are entangled in the causes and consequences of the destruction of life, and the ecosystems which support life, on our planet [1]. As Stephens writes with respect to climate justice, while higher education systems should be well positioned to accelerate societal transformation, many institutions and funding pathways are constraining diverse ways of thinking and knowing within ‘narrow legacy disciplinary boundaries’ [2]. We draw attention to our ‘narrow legacy disciplinary boundaries’ in vector-borne disease research, and the space that it erases, to think about vector-borne disease, in a different way amid rapid global change.

We first evidence how European vector-borne disease research played a role in colonial expansion in Africa, focussing on malaria and sleeping sickness; two diseases that were a problem for colonialists, and with which the authors have experience. Drawing on this history, we show the origins of a dominance of a biomedical approach to vector-borne disease control in tropical medicine, and consequently global health. European knowledge systems heavily influenced research in Africa, a geographical area of focus in global health because of colonial ties from tropical medicine to dominant institutions in the Global North and because of persisting health inequities.

We then critique the resulting negative impacts of commodities for control, funding inequities, disciplinary silos and the need for an epistemic shift. In seeking an epistemic shift, we look to Latin American scholarship on social medicine. Perez-Brumer *et al.* [3] propose that because of earlier independence from colonialism in Latin America, compared with Africa or Asia, it was possible to develop infrastructure to support their own forms of knowledge production. In contrast to biomedicalization in Europe, a strong emphasis on social medicine developed [4]. At the same time, however, as global health expanded in the 2000s, Latin America’s influence in the field declined for a variety of reasons relating to decision making predominantly by Global North institutions [3], thus maintaining the dominance of European approaches developed during the colonial era.

We demonstrate how scholarship from social medicine and across social, ecological and environmental sciences alongside related scholarship on decoloniality show alternative paths. Our hope is to expose readers to literature not often covered in Western disease ecology and epidemiology. Latin American epidemiologists have been calling out the limitations of Western epidemiology and the need for a different paradigm, for at least 50 years [4]. We hope readers will be encouraged to dive into our list of references and join in reflecting on what it should mean to engage with historically situated disease research, within the fields of global [5] and planetary [6] health.

## Historical interventions against malaria and sleeping sickness in Africa

Mavhunga [7] describes how, before European colonialism, ‘strategic deployments’ were used across kingdoms in Africa to reduce contact with tsetse flies, the vectors of sleeping sickness. These strategies included leaving certain areas unsettled, and organised nightly or seasonal livestock movements. Importantly, Mavhunga [7] states that before the arrival of European colonialists, “…*vatema’s (black, also vanhu – peaceful people) approach (to sleeping sickness) control was rooted in a deep spiritual ethic toward earth’s endowments, not a lack of means of mass destruction*”. Indigenous practices have also been detailed for malaria [8] like predicting the onset of malaria season using plant and insect phenology, using plants and smoke to repel mosquitoes, and herbal medicines as a variety of approaches likely handed down generationally for malaria control. Packard details how malaria was often common but nonfatal prior to colonialism [9]. As we evidence below, European colonialism inflicted an interruption of Indigenous practices and of the natural and social conditions that may have kept infectious diseases such as sleeping sickness and malaria in check.

In his book concerning the atrocities in the Congo [10], Hochschild details how, due to colonialism, more Congolese died from disease than directly from bullets. Indeed, Laveran, Mesnil and Nabarro, in 1907 [11] wrote with respect to sleeping sickness in the Congo ‘*the mortality which in 1896 was 13% and in 1897 19% rose in the following years to 39% and 22% respectively, and in the first quarter of 1900 to 73%. The majority of children died of the disease…Todd shows that the enormous spread and great increase in sleeping sickness in the Congo basin have been due in great measure to the increase in travel following the opening up of the country…with the advent of white men, new steamer and caravan routes were opened up, with the result that the infected areas have increased rapidly in number and extent.*’ In addition, in the report of their expedition to the Zambesi, in the region at the time named Rhodesia and, occupied by the British South Africa Company, Kinghorn and Montgomery [12] document how sleeping sickness likely spread from Katanga mines in the Congo Free State across northeast Rhodesia ‘*as labour was not drawn from one particular district*’.

Similar circumstances have been documented for *Anopheles* and malaria. For example, a Liverpool School of Tropical Medicine (LSTM) malaria report from an expedition to Nigeria [13], describes how colonial activities created *Anopheles* larval habitats: ‘…*Between the factory (of the Niger Company, a British mercantile company) boundary and the native huts we discovered a typical Anopheles pool containing numerous larvae. The pool was a portion of a very badly constructed drain which ran through the factory’s area, which in its course also afforded several other breeding-places*’. It also mentions larval habitats created by the construction of roads and footpaths and the report states that *‘these samples serve to illustrate how the unhealthy condition of a European settlement is brought about often by the Europeans themselves… by the unwitting neglect of elementary sanitary and engineering principles*’.

Between 1900 and *c.* 1970, the decline of malaria and elimination of the disease as a public health problem in Europe correlated with increases in Gross Domestic Product (GDP), life expectancy and urbanization [14]. While specific interventions against malaria, including larviciding, drainage and the use of quinine likely also contributed, there is evidence that socio-economic factors were the main driver of elimination in Europe^76^. In many parts of sub-Saharan Africa, malaria grew as a problem over the same period through which it was eradicated in Europe. This trend has been attributed, at least in part, to colonial activities which caused land dispossession, altered ecosystems and biodiversity loss, changes to agricultural practices, labour conscription, forced migration and increased population densities [15].

These colonial conditions exacerbated vector-borne disease, as they destroyed social structures and changed land use, and are the very conditions within which Western vector-borne disease research was conceived. Hochschild [10] refers to medical knowledge as a tool which allowed Europeans to seize most of tropical Africa from the late 1800s. Malaria and sleeping sickness – among other diseases – were explicitly controlled to reduce the high death rate experienced by white people in Africa and elsewhere. Indeed, the first expeditions and research of tropical medicine institutions (LSTM, London School of Hygiene and Tropical Medicine, Imperial College London) were economically motivated and funded by merchants who profited from extractivism [16]. Bump and Aniebo [17] elaborate on how malaria became a threat to military control and colonial ambitions and was therefore a key focus for study and control to enable further extraction of resources. For both malaria and sleeping sickness, the solutions to control during colonialism were hierarchical. For malaria, segregation and vector control were decided on as the solution rather than the more expensive efforts to improve sanitation [18]. For sleeping sickness, control measures also involved segregation, specifically of people who were suspected of being infected with trypanosomes.

During the expedition to the Congo Free State with money given to LSTM by King Leopold, scientists Christy, Dutton and Todd observed and performed medical procedures on Indigenous people with symptoms of sleeping sickness. The recommendations by Dutton and Todd from the Congo expedition were then used in other colonised parts of Africa. In their report from the Zambesi expedition between 1907 – 1908, Kinghorn and Montgomery write ‘*we have used the classification adopted by Dutton and Todd…9005 natives examined, 1878 had palpable glands…in as many cases as was practicable gland puncture was performed…*’. They then report that ‘…*we are in a position to weed out the infected and isolate them before they become dangerous*…’. They list the ‘*control of native movements’* as one measure and recommended *‘a sufficient number of special medical officers be appointed to travel systematically through all villages palpating all the natives and puncturing those in which this was indicated. In the event of cases being discovered, they should at once be removed to a segregation camp for treatment’*. In the report, the motives for these measures are clear: ‘…*for as you will see, the* *success of this territory, commercial and industrial, will be extensively modified should good come of any preventative or curative measures’* [19]. As summarised by Headrick (2014) [20] people in camps were given atoxyl; camps involved ‘*…painful treatment, poor conditions, lack of food, and permanent separation of patients from their families. To prevent the sick from escaping, they had to be guarded by soldiers’*.

Hence, for both sleeping sickness and malaria, a focus on controlling the parasite and vector became the dominant task in tropical medicine to aid colonialism without regard to Indigenous people. It was based on a racist ideology. While racism may not be as overt in research today, the narrow focus with respect to control strategies has persisted, as have colonial legacies of disciplinary silos and power inequities in funding and leadership.

## Current control strategies

The WHO reports that the use of commodities such as insecticide-treated nets (ITNs), indoor residual spraying of insecticides, and drug treatment to protect against malaria has averted an estimated 2.2 billion cases and 12.7 million deaths from malaria since 2000 [21]. In 2023, 47 of the 83 malaria-endemic countries worldwide reported <10,000 cases of the disease and four countries were certified malaria-free: Azerbaijan, Belize, Cabo Verde and Tajikistan [21]. Successes have also been reported for sleeping sickness. Between 1999 and 2023, the total reported number of new cases of Gambian sleeping sickness fell by 98% from *c.* 30,000 to <1000. Over this period, the use of screening and treatment with drugs, combined with tsetse control using insecticide-treated targets (ITT), has contributed to this success. Estimates suggest a reduction of *c.* 25% in cases in north-western Uganda between 2012 and 2019 because of ITT [22]. Along with case detection, ITT are estimated to have reduced new infections per year from *c.* 300 in 2013 to a mean of 7 and 0 in 2014 and 2015 in the Mandoul focus, DRC [23]. Further, no native cases were detected in Burkina Faso between 1993 and 2015 [24], while the country used to experience hundreds of cases before the 1980s [25].

For both malaria and sleeping sickness, these efforts have required extensive coordination of the funding, manufacture and distribution of products, to ensure their timely delivery and for nets and targets, their replacement. For example, Burkina Faso – with an estimated 8.1 million malaria cases in 2023 [21] – organised to deliver 17 million ITNs to an estimated population at risk of *c.* 30 million people in 2025. Commissions are set up at the Central, Regional, District, health training and village levels to manage the process, taking responsibility for storage, security, logistics, training and delivery. Insecticide-treated nets are produced predominantly by companies registered in Europe and Asia and shipped into the country roughly three months in advance by the US President’s Malaria Initiative (PMI) (a programme under the now defunct USAID).

In the report of the 1950 Malaria Conference [26], three key aspects of control were apparent: i) many breeding sites of *An. gambiae* were created by ‘human activity’ (of which the part attributed to colonial activities was not discussed); ii) that it was dangerous to assume that experience gained in one place can be applied effectively in another; and iii) the widespread use of insecticides must be implemented with caution due to the potential detrimental impacts on pollinator insects and food production. The application of commodities for vector-borne disease control has arisen, in part, from their colonial origins, but also from their ability to be deployed across contexts and the desire to tell a compelling story to funders, with commodities being easily associated with impact, unlike other priorities including strengthening health systems management [27]. The desire to tell a compelling story has perhaps inhibited nuanced critique of biomedical dominance in vector-borne disease research, exacerbated by power being held predominantly in institutions founded during the colonial period where biomedical dominance was established. This lack of critique includes grappling with the potential negative impacts of large-scale deployment of commodities. In the next section, we focus on the negative impacts of mosquito nets, but many of the points made are relevant to other commodities used in health and beyond.

## Commodities and the pollution they create

The first versions of ITNs could be re-dipped into insecticide periodically. However, these nets are now generally manufactured using either a polyethylene or polyester plastic with insecticide either incorporated into or coated onto the plastic fibres and designed to last for three years [28], though often durability estimates are lower [29]. The widespread use of ITNs has led to increased plastic production and disposal, raising environmental problems [30]. As millions of long-lasting, polymer-based nets are discarded annually [31], waste management becomes more urgent. Both urban and rural communities in malaria-endemic Africa often lack management strategies for municipal waste, having – historically – not required this service. Generally, structured collection or environmentally safe disposal of expired or damaged ITNs is not costed into ITN campaigns. Disposal methods such as burning, landfilling, or repurposing are undertaken instead and pose risks to both ecosystems and human health.

Plastics are a bi-product of oil meaning that the carbon-based economy benefits from their use: the global plastics market size was $579.7 billion in 2020 – 100x more than the funds invested into malaria control annually – with an expected compound annual growth rate of 3.4% from 2021 to 2028 [32]. The plastics industry is responsible for 1.8 billion tonnes of greenhouse emissions per year and, in 2019, 22 million tonnes of plastics leaked into the environment globally [33]. As of 2020, the healthcare plastics market was worth US$22.26 billion, or 2% of total plastics production by value, and is growing by 6.1% per year [34]. There is growing concern around the volume of waste generated through universal ITN delivery and it is estimated that dual active ingredient long lasting ITNs manufacturing alone will produce 57,500 tons of plastic annually by 2030 [35]. Thus, rising plastic waste production is increasingly visible in malaria-endemic areas, where discarded ITNs contribute to pollution and environmental damage. As malaria control efforts grow, managing plastic waste from ITNs and other commodities such as spatial emanators, poses a significant problem for already burdened communities.

Pollution at the end of use life of ITNs occurs through various routes. The environmental impact of ITN disposal poses significant problems for communities and policymakers, with waste management often left to local populations lacking sufficient support. In Burkina Faso, a 2023 survey identified clear regional differences in ITN disposal; burning or burying were most prevalent in the Sudanian zone, while in the Sudan-Sahelian and Sahelian zones old ITNs were most often adopted for alternative use [30]. In Tanzania, burning and landfill were also the most common endpoints for old ITNs [36]. Burning ITNs can release toxic fumes, including dioxins [37] and landfill, if not properly sealed, leaches plastic into the environment as it degrades [38]. Plastic pollution makes a major contribution to the novel entities’ planetary boundary [39], taking a long time to degrade, and invariably ending up as very small particles. Nanoplastics – measuring 1 nm up to 1 μm – and microplastics, 1 μm to 5 mm, can enter our body through inhalation, consumption, or even penetration through skin and are particularly prevalent in cosmetics and clothes [40]. These plastics bioaccumulate and can trigger respiratory disorders (lung cancer, asthma and hypersensitivity pneumonitis), neurological problems (fatigue, dizziness), and inflammatory bowel disease; studies also report that nano- and microplastics can cause cell death and have both genotoxic and cytotoxic effects [41]. There is now, in negotiation, a global plastics treaty, as the UN are calling for regulation on plastics. The debate is currently at an impasse. The reason is that fossil fuel producing countries do not want this treaty to bring regulations on production [42]. Other countries want regulation on both the supply and demand sides of the plastics industry. The reasons are clear, primary plastics come from petrochemicals and demand for the traditional use of these (energy and fuel) is declining [43], and these industries are eyeing plastics as the way out of losing that market.

Without acknowledging these wider dynamics, and how commodities in health relate, as health-related scientists we are contributing to the *status quo*. Whilst the dominant approach may reduce disease-specific morbidity and mortality, we are missing the bigger picture. If we are only tackling a symptom with a commodity, rather than the root causes at the same time, we are only achieving temporary gains, that require episodic action; our efforts inadvertently contribute to the climate crisis and biodiversity loss, subsequent health impacts and continue to turn a blind eye to an extractive economic system [44]. We argue that contributing to maintain this dominant approach are colonial legacy inequities in funding in addition to disciplinary silos.

## Legacy inequities in funding and disciplinary silos

There is an increasing literature calling out problems in global health and specifically vector-borne disease across funding, research and implementation. This includes the bias toward funding research and production in Global North countries and the narrow focus on biomedical interventions, due to legacies of colonialism within and outside of global health [45–48], as we also document here.

Baird (2017) [49] raised the alarm about an over-reliance on commodities instead of malaria prevention that considers environmental alterations to reduce the anopheline mosquito population, and raised the question ‘*when commodities vanish, how to then deal with an unresolved malaria problem?*’. In 2025, the ramifications of a major donor removing itself from the story unfolded, as the current US administration cut international aid. In 2017, Winskill *et al*. [50] explored the consequences of the removal of financial support from PMI, finding that a 44% reduction in PMI funding could result in an additional 290,649 (95% CrI: 167,208, 395,263) deaths between 2017 and 2020. Around 5,800 contracts under USAID (90% of its work) have been revoked under the Trump Presidency, including a $90 million Chemonics contract for malaria prevention, affecting 53 million people [51]. Recent estimates of the impact of freezing funds for PMI indicated a likely 14.9 million (12.5 M - 17.8 M) additional malaria cases and 107,000 (71,000–166,000) additional deaths from January to December 2025 [52].

The funding structure for malaria control is generally based on supporting the use of preventive interventions recommended by the WHO, an organization serving over 90 countries, so the advice it provides is necessarily generalised. The intervention tools that pass are correspondingly generic as they have been able to accrue sufficient (multiple) cluster randomized trial evidence in most settings [53,54]. Meta-analysis of these state-of-the-art trials are rightly considered the highest quality of evidence. A concern is that this approach limits the adoption of tailored, context-specific interventions and other approaches including larval source management or educational programs where impacts may be powerful in certain contexts but limited in others [55]. To secure essential interventions, countries negotiate with donors. Donor organizations can support both core and supplementary interventions that are recommended by WHO but after the negotiation process, the implemented strategies are dominated by provision of core interventions. For example, as a leading donor, The Global Fund, reports the supply of over 334 million parasitological tests, nearly 171 million treatments, over 227 million ITNs, over 44 million children receiving seasonal malaria chemoprevention and nearly 8 million households receiving indoor residual spraying in 2023 [31]. This vast support is offering critical impact, but this structure potentially inhibits more tailored, context-specific and co-created strategies.

Barriers to addressing the above limitations nearly always include funding, but also decision-making structures, and the levels that we wish to organise within. In an open letter, Erondu *et al.* (2021) [56] emphasised the point that while progress has been made on malaria control, it is limited by the fact that funding structures often diminish the capacities and ownership by individuals and organisations in the countries affected. The authors drew attention to US$ 30 million awarded by the US President’s Malaria Initiative to institutions in the USA, UK and Australia for malaria research, even though there are leading institutions with expertise in this area in Africa, South America and the Indo-Pacific Regions, where the disease is rampant. In addition to funding decisions, Okumu *et al.* (2022) [57] warn against a narrow commodity-driven vision of disease control. Relying strongly on commodities such as ITNs encounter difficulties in certain contexts. For instance, level of education, social position and religion can affect rate of mosquito net use observed in certain localities or regions [58]. Thus, there is a need for ownership to lie with affected communities including local workforce employment, knowledge, trust and agreement on actions [57].

Our shared histories and the planetary crisis are entangled in the hoarding of wealth [44] and require of scientists the ability to critically reflect on our role in maintaining unjust power structures. There are only two generations of scientists, who handed down a copy of Trypanosomes and Trypanosomiasis [11], between T.A.M. Nash and JSL. Our current approaches have been inherited from a patriarchal, racist, colonial system which did not strive for transdisciplinary research carried out with communities, that could also contribute to social, political and environmental transformation [4].

On climate justice, Stephen’s [2] writes ‘…*This technocratic approach reinforces climate isolationism, the term I have coined to describe the narrow way of framing climate change as a scientific problem requiring technological solutions…By framing climate change as an issue that is separate and disconnected from other social issues, climate isolationism upholds the* *status quo systems by encouraging non transformative ideas to reduce the problem…it is dangerous because all too often research attempts to treat the symptoms rather than the cause*.’ This could be re-read, swapping ‘*climate*’ for ‘*disease*’. Whether a scientist in the early 1900s, or a scientist today, similar processes are occurring and often the same narrow focus is taken by Western science, whether it be disease isolationism or climate isolationism.

Specialised tech-solutions offer immediate support but generally have implications in the longer term (environmental waste, reliance on undisrupted distribution channels [50], insecticide resistance) rendering these efforts sub-optimal. In addition, ethnographic research concerning *Aedes*-borne disease has demonstrated how ‘mosquito-centrism’ obscures the social and embodied experiences of epidemics [59]. Top-down generalised approaches to vector control also often result in unseen local impacts on those involved in the labour of such activities, including adverse health impacts of chronic exposure to insecticide [60] and experiences of violence, highlighting workers awareness that mosquito-borne diseases are only a small fraction of the plethora of issues faced by the communities where these diseases are endemic [61].

Our continuation of an approach where technology dominates, is tied to disciplinary silos that were developed during colonialism [4]. Yes, technology and innovation are essential, but we also must recognise the reality of global health origins in colonial tropical medicine, interconnectivity between our agricultural and industrial systems and health outcomes, and restricted ecological considerations for vector-borne disease research as a grounding to properly value alternative approaches.

Researchers are drawing attention to limitations of a dominance of biomedicine in health research [62,63]. This dominance contributes to a continuation of local and global inequities and neglects root causes like extractivism, which are, in turn major contributors to disease burden and poor health outcomes [64]. The compartmentalised structure of research essentially sets epistemic limits to the understanding of why some populations suffer greater burden of disease than others [65]. Importantly, Perez-Brumer *et al*. [3] evidence how this narrowing of global health has been linked to the marginalisation of scholarship from Latin America, but that it is this scholarship which offers important lessons for the next iterations of global heath. We elaborate on this in our last section alongside other scholarship that provides a potential guide for broadening research.

## Paths forward

While acknowledging the achievements made in infectious disease research and control, for malaria there were still an estimated 282 million cases and 610,000 deaths worldwide in 2024 – about 9 million more cases than reported in 2023 [66]. Approximately 94% of deaths occurred in the WHO African Region (47 member states, not including Djibouti and Sudan) where many people at risk still lack access to the services they need. Controlling vector-borne diseases contributes to improving health and reducing the impacts of poverty caused by these diseases. Pursuing this goal, however, continues predominantly through a conveyor belt of relatively short-lived commodities [67], many without a circular life cycle, and with adverse environmental impacts.

Our aim is not to promote the idea that communities should be denied commodities that protect against vector-borne disease – far from it. Rather, alongside greater attention to alternative approaches as detailed below, we ask those involved in product development and procurement for vector control to take responsibility for the full impacts of their products and factor in wider consequences during their design and implementation. By doing this, those working in product development could and should be leading the way that products, more broadly, need to be reduced or recycled. For ITNs, arguably still an essential use of plastic, organizations exist to directly address some of these environmental problems [68] and companies are focussing on offering possible solutions. Countries and donors are requesting that ITNs are no longer individually wrapped but instead distributed as bales of 40 or 50 ITNs per bundle [69] and this has offered a substantial reduction in overall waste given individual packaging contributes around 0.01 kg CO2_e_ per net. There are now 100% closed-loop recycled ITNs [70] (though collection schemes for old nets need to be established, and these products currently remain more expensive than using primary polymers for ITNs), and plastic bricks are being developed in Nigeria using end-of-use-life ITNs [71]. Despite these efforts, ultimately, to reduce reliance on the use of commodities, alongside necessary changes to decision-making and funding structures [56,57], an epistemic shift is required. Latin American social medicine has been marginalised in global health research [3], but here there already exist alternatives to the dominant culture of disease isolationism.

In a detailed review, Waitzkin *et al*. [72] summarise Latin American scholarship concerning social medicine, which as a foundation assumes populations and social institutions are more than the sum of the individuals comprising them [73]. As example, Breihl defines the ‘social determination’ (as opposed to social determinants) of health as subsumption of individuals by societal processes including social reproduction by capital accumulation, political and cultural relations, with individuals’ having greater or lesser autonomy to respond [4]. He then situates this dynamic alongside the metabolism of nature under our current capitalist global economic system which together results in a dialectical process influencing a health-illness spectrum [4]. In social medicine and critical epidemiology, focus is therefore often on social class, economic production and culture rather than just individual characteristics [74]. This therefore leads to studies focussed on the health impacts of capitalism, imperialism and multinational corporations [72].

Critical epidemiology as applied to dengue research, for example, explains the production of risk as involving the territorial expansion of agro-industrial extractivism resulting in conditions favourable for *Aedes* reproduction and worsening of regional social class gaps creating collective patterns and conditions prone to higher dengue transmission [4]. Responses by researchers have been broader than chemical-based vector control, including involvement with communities to implement educational programmes in schools, community participatory larval source reduction efforts with government activities and importantly the empowerment of community members to demand infrastructural change from governments that would also result in wider health benefits beyond vector control [75].

In line with a social medicine approach, Okumu *et al.* (2022) [57] outline how there is a need to gradually reduce overreliance on commodities for malaria in Africa while expanding multisectoral initiatives such as improved housing and environmental sanitation and health systems strengthening. Shifting consideration of actions toward infrastructural changes should be prioritised where possible [76]. This would offer wider benefits to health beyond just vector-borne disease with matching (or improved) efficacy, and ecological protection. Between 2000 and 2015, the proportion of Africans living in improved homes had grown by *c.* 22% and most of these were funded from household incomes [77]. The disconnect between disease control and living environments is brought about only when scientists and policy makers start excessively championing the use of commodities, without mentioning the importance of improved environments (or even management of agricultural systems) in building resilience. This requires a different set of skills and knowledge, beyond laboratory techniques and statistics, including local contextual knowledge [78] and an ability to work within a transdisciplinary environment. It requires greater emphasis on connecting communities, experts across different disciplines and other stakeholders.

Other approaches being implemented in malaria research include housing modifications that address both heat stress and vector biting [79] and community larval source management [80]. In these examples, the importance of investing in locally based research leaders who have close relationships with communities and an understanding of the bio-eco-social context seems obvious, but this is often still not the norm [64]. To achieve meaningful progress in health and environmental issues, open dialogue and respect among policymakers, scientists, and affected individuals are essential. Participatory and co-created research and interventions hold promise to foster such relationships and to broaden approaches [81]. However, in a systematic review of malaria interventions including community engagement between 2000 and 2022 most studies were only intended to inform and involve, with just seven of the total 75 studies being collaborative and only one co-led with community stakeholders [82]. While the authors recommended that national programmes must work with community members to develop ownership and work towards co-leadership, this is a complex process, with the necessity to attend to the power dynamics involved [83]. Using the social ecology of power framework [84], Egid *et al*. assessed how power influences participatory health research and provides a set of tools and techniques to address power inequities across partnerships [83]. As part of this process, it must be considered how the perceived importance of vector-borne disease risk differs relative to other concerns and priorities, at the household, community and wider health system levels, again necessitating that vector-borne disease research and control be fitted into wider priorities and agendas across multiple social scales.

To implement these changes, a shift in leadership away from the Global North is overdue, enabling targeted efforts that are more accepted by recipient communities given the increased trust in those delivering efforts. In calling for change, Besson *et al.* (2022) [45] provides a guide for identifying epistemic injustice in global health research funding practices and Oti *et al*. (2024) [85] provide a framework for institutional allyship by global health funders. Amongst other important points, they highlight the necessity for increased funding to community-based organisations in the Global South with fewer restrictions over how funds are spent, and the need to address limitations that occur from a demand for research to be generalisable/ decontextualised.

For those working in global health institutions in the Global North, distant from where their foreign gaze [64] has been inherited to fall, what then? We live in a global economic system. We suggest a role there to connect disease risk to corporate determinants of health and quantify transnational commodity supply chain drivers of health risks, including vector-borne disease. In this respect, a particular role for Global North researchers could be to develop more collaborations with groups and organisations that hold social justice and climate proactive ethics at their core. This would enable greater attention and contribution toward governance and legal routes [86] to accountability for corporations, wealthy countries and communities in upholding inequitable global systems. For example, the interaction between climate change, migration and unplanned urbanisation in the context of expanding tropical commodities demanded by corporations generates questions on the relationship between agrarian socioeconomic and political systems and the spread, effects and remediation for tropical diseases, including those that are vector-borne. These interactions are part of transnational supply chains that involve the actions of individuals in one part of the world affecting the lives of many in another [87]. Between 2016 and 2018, *c.* 21.3 million hectares were required annually to supply the UK’s demand for seven soft commodities; an increase of 15% compared to 2011 – 2015 [88]. The UK’s overseas land footprint continues to expand, with palm oil, rubber and cocoa, three of the seven key soft commodities, with resulting land use change and widening wealth inequality likely playing a role in changing vector-borne disease risk.

Vector-borne diseases are woven through a colonial history, connecting ecosystems and human health, continuing to be exacerbated by extractivism and other forms of exploitation. By using some of the history of our area of research, we have shown how vector-borne disease research is political and inherited. Lewontin and Levins wrote: ‘*We believe that science…is a social process that both causes and is caused by social organization. To do science is to be a social actor engaged…in political activity. The denial of the interpenetration of the scientific and the social is itself a political act, giving support to social structures that hide behind scientific objectivity to perpetuate dependency, exploitation, racism, elitism, colonialism’* [89].

We need to have specialists in vector-borne disease, and specialists in other areas of epidemiology and health, to continue addressing the symptoms of our dominant culture of extraction. However, we also need to urgently make more space for other approaches to health which don’t compartmentalise in the same way, and for generalists, who can bridge across disciplines and work together across cultures to address root causes [4]. We need more scientists trained in critical theory [90]. Other researchers are already showing a different path, and we refer to literature including Breihl [4], Abimbola [64] and Stephens [2].

For the self-reflective scientist, questions arise about another way of doing science, of knowing the world, how to critique what is inherited and how to start to find ways of doing things differently, so we are careful what we pass on to the next generation. In his paper ‘East Africa: Science for Development’, Odhiambo (1967) [91] states ‘*African philosophy is a monism (a one-world view) while Western philosophy is a dualism (a subjective as well as an objective world)… monism has deprived him (the African) of the choice between either science or mysticism; instead he has concentrated his intellectual powers in devising a vastly intricate social and communalistic system’…*and he calls to *‘… reach the basic root of the problem, his monistic world-view, and can modify it in a manner which he can begin to regard Nature apart from himself or other beings*’. Yet it is at the same time Western dualism, the illusion of separation from nature, in which Western science is rooted, which has facilitated colonialism and the climate and nature crises, and which now makes it hard for Western scientists to address. We are caught up in the process of a destructive kind of progress.

Mirroring the words of Odhiambo, as individuals embedded in Western scientific systems, we need to reach our basic root of the problem, our dualistic world-view, and modify it in a way we can begin to regard nature as part of ourselves and ourselves as other beings. Therefore, we suggest that these two opposing forces; monism and dualism work with each other to create and re-create our worlds. There is a need to pay attention to, and balance both. The role of governments should be to protect the people and environments that they serve; our role as scientists is to generate the knowledge that can allow political decisions to be made in our collective best interests. This system has failed, partly due to pressures on governments from those singularly focused on economic endpoints, partly due to a general mistrust of experts through misinformation on social media and other platforms. Part of this lethargy is because people feel unheard. Honestly recognising our social, scientific and inequitable history has potential to recover some of this lost trust. We need scientists and health researchers who are prepared to engage in political discussions and work alongside campaigners and community members who are working toward health justice and for a liveable planet [92].

## Conclusions

Current vector-borne disease control relies on a narrow, biomedical approach that reduces deaths but ignores root causes like inequality, environmental drivers of transmission, and ecosystem changes that can result from control practices. As such, diseases are often treated as separate technical problems, excelling at symptom suppression while perpetuating colonial legacies and masking interconnected social-ecological drivers, which we refer to as disease isolationism. As a technical response, the heavy use of bed nets, insecticides, and drugs saves lives, yet produces massive waste and reliance. New intervention strategies must reduce all forms of environmental harm and promote local resilience, alongside health benefits. We ask for the recognition that broader strategies are constrained by colonial-era power imbalances which require reflection, freeing endemic countries to determine their own targets. Sustainable vector control requires integrated, decolonial strategies co-designed with communities, that address social justice, planetary health and long-term sustainability.
